# Differential Splicing Alters Subcellular Localization of the Alpha but not Beta Isoform of the MIER1 Transcriptional Regulator in Breast Cancer Cells

**DOI:** 10.1371/journal.pone.0032499

**Published:** 2012-02-24

**Authors:** Jaclyn A. Clements, F. Corinne Mercer, Gary D. Paterno, Laura L. Gillespie

**Affiliations:** Terry Fox Cancer Research Laboratories, Division of BioMedical Sciences, Faculty of Medicine, Memorial University of Newfoundland, St. John's, Newfoundland, Canada; Russian Academy of Sciences, Institute for Biological Instrumentation, Russian Federation

## Abstract

MIER1 was originally identified in a screen for novel fibroblast growth factor activated early response genes. The *mier1* gene gives rise to multiple transcripts encoding protein isoforms that differ in their amino (N-) and carboxy (C-) termini. Much of the work to date has focused on the two C-terminal variants, MIER1α and β, both of which have been shown to function as transcriptional repressors. Our previous work revealed a dramatic shift in MIER1α subcellular localization from nuclear in normal breast tissue to cytoplasmic in invasive breast carcinoma, suggesting that loss of nuclear MIER1α may play a role in breast cancer development. In the present study, we investigated whether alternative splicing to include a cassette exon and produce an N–terminal variant of MIER1α affects its subcellular localization in MCF7 breast carcinoma cells. We demonstrate that this cassette exon, exon 3A, encodes a consensus leucine-rich nuclear export signal (NES). Inclusion of this exon in MIER1α to produce the MIER1-3Aα isoform altered its subcellular distribution in MCF7 cells from 81% nuclear to 2% nuclear and this change in localization was abrogated by mutation of critical leucines within the NES. Treatment with leptomycin B (LMB), an inhibitor of the nuclear export receptor CRM1, resulted in a significant increase in the percentage of cells with nuclear MIER1-3Aα, from 4% to 53%, demonstrating that cytoplasmic localization of this isoform was due to CRM1-dependent nuclear export. Inclusion of exon 3A in MIER1β to produce the N-terminal variant MIER1-3Aβ however had little effect on the nuclear targeting of this isoform. Our results demonstrate that alternative splicing to include exon 3A specifically affects the localization pattern of the α isoform.

## Introduction

MIER1 is a fibroblast growth factor (FGF)-activated transcriptional regulator [1] that is highly conserved in evolution, with 95% identity between human [2], [3] and mouse sequences [4] at the amino acid level. The *mier1* gene produces multiple distinct transcripts through exon skipping, facultative intron usage, alternative polyadenylation sites and the use of two alternate promoters, P1 and P2 [3]. The resulting MIER1 proteins share a common internal region, but vary in their amino (N-) and carboxy (C-) terminal sequences. To date, we have cloned and characterized isoforms with three distinct N- and two distinct C- termini in humans [3], although additional variants have been reported in GenBank.

**Figure 1:**
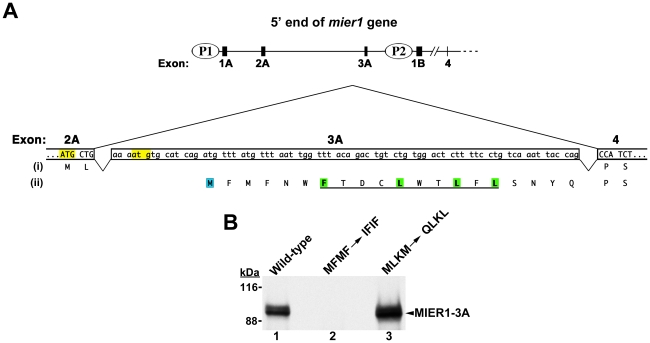
Sequence encoded by exon 3A. (A) Schematic illustrating the 5′end of the *mier1* gene and the nucleotide & predicted amino acid sequences of exon 3A. The upper diagram shows the location of exon 3A relative to the 2 promoters, P1& P2. The lower diagram shows the sequence of exon 3A and illustrates alternative splicing to generate either *mier1* (exons 2A+4; protein sequence (i)) or *mier1-3A* (exons 2A+3A+4; protein sequence (ii)). The MIER1 start codon, located at the end of exon 2A, is highlighted yellow, as is a downstream in-frame ‘atg’. For the analysis in (B), both highlighted ATGs were mutated to avoid the possibility of spurious translation initiation at the second ‘atg’; this double mutation changes the putative protein sequence from MLKM to QLKL. The predicted start methionine for MIER1-3A is highlighted in blue; the consensus NES is underlined and the hydrophobic residues are highlighted in green. For the analysis in (B), both the initiation and third codons were changed to produce an MFMF->IFIF mutant. (B) Effect of mutating the putative initiation codons on *in vitro* translation. ^35^S-labelled rabbit reticulocyte lysates programmed with wild-type (lane 1) or either of the two mutant *mier1-3A* cDNAs (lanes 2–3) were immunoprecipitated with anti-MIER1 and analyzed by SDS-PAGE and fluorography; the position of full–length MIER1-3A is indicated by an arrowhead and the molecular weight standards are shown on the left. Mutation of the predicted initiation codon (MFMF->IFIF) located within exon 3A abrogates production of full-length MIER1-3A (lane 2), while mutation of upstream ATGs (MLKM->QLKL) has no effect (lane 3), thus confirming the N-terminal sequence of this isoform. Note that full-length MIER1 proteins migrate aberrantly on SDS-polyacrylamide gels, as has been reported for other proteins containing stretches of acidic residues [38], [39].

The N-terminal isoforms result from differential promoter usage and alternative splicing [3] (Fig. 1A). Transcription from P1 or P2 produces mRNAs with distinct 5′UTRs, but the resulting protein sequences (named N2 and N3 in GenBank) are identical except for the first two amino acids after the start methionine. A third N–terminal isoform arises only during transcription from the P1 promoter, which allows for alternate inclusion of a cassette exon, exon 3A; doing so produces a variant containing additional sequence at its N-terminus (named N1 in GenBank). The two C-termini differ significantly in size and sequence [3]: the α C-terminus contains 23 residues and arises by removal of a facultative intron during RNA processing, while the β isoform contains 102 residues and results from inclusion of this intron with subsequent read-through translation to a stop codon within the intron.

All of the experimental research to date has focused on the MIER1α and β C-terminal isoforms that lack the cassette exon 3A sequence at their N-terminus. While no specific function has been assigned to the β C-terminus yet, it contains a strong nuclear localization signal (NLS) [3], [5]. An additional weak, cryptic NLS has been identified in the internal common region of MIER1, however only the β C-terminal NLS was shown to be necessary and sufficient to target MIER1 exclusively to the nucleus [5]. One would therefore predict that MIER1β is nuclear, while MIER1α is localized exclusively in the cytoplasm. However, we have demonstrated that MIER1β is sometimes retained in the cytoplasm, e.g. during early *Xenopus* development [6] and that MIER1α isoform is occasionally targeted to the nucleus, e.g. in human mammary ductal epithelial cells [7] and some cell lines (unpublished observations). Thus, the NLS-dependent nuclear/cytoplasmic distribution of MIER1 is not absolute, but can vary with cell type and stage of differentiation.

The common internal region of MIER1 contains several domains important for its role in regulating transcription [1], [8]–[10]. These include an N-terminal acidic region, which can function in isolation as a transcriptional activator [1]; an ELM2 [11] domain and a SANT [12] domain. ELM2 and SANT domains have been found in a number of transcriptional co-repressors, including NCoR [13], SMRT [14] and members of the MTA family [15]. In MIER1, the ELM2 domain has been shown to recruit histone deacetylase 1 (HDAC1) and repress transcription [8]; the SANT domain interacts with Sp1, displacing it from its cognate binding sites in the promoter of regulated genes, including MIER1's own promoter, and interfering with transcription [9]. In addition, MIER1 has been shown to bind CREB-binding protein (CBP) and inhibit its histone acetyltransferase (HAT) activity [10]; the CBP interaction domain has been mapped to the N–terminal region that includes both acidic and ELM2 domains [10]. MIER1 also interacts with the G9a histone methyltransferase [16], however its effect on the activity of this chromatin modifying enzyme has yet to be determined.

The MIER1α-specific sequence contains a classic LXXLL motif [17] for interaction with nuclear hormone receptors. Indeed, we have shown that MIER1α interacts with ERα in MCF7 cells and that regulated overexpression inhibits estrogen-stimulated anchorage-independent growth [7]. Furthermore, analysis of the MIER1α expression pattern in breast samples from patients revealed a dramatic shift in the subcellular localization in ductal epithelial cells, from nuclear to cytoplasmic, during progression to invasive carcinoma [7]. These data suggest that controlling nuclear levels of MIER1α may play an important role in the development of invasive breast carcinoma.

Further examination of the sequence encoded by the cassette exon 3A reveals the presence of a putative nuclear export signal (NES) and therefore, alternative splicing has the potential to regulate the subcellular localization of MIER1α. In this report, we have investigated the ability of the exon 3A sequence to function in nuclear export and have determined its effect on the subcellular localization of the MIER1α and β isoforms.

## Results and Discussion

### Verification of the MIER1-3A initiation codon

In our original report describing the cloning and characterization of human MIER1, the N–terminal variants were given the non-descript names N1, N2 and N3 [3]. For clarity and ease of understanding, we have renamed the variant containing exon 3A, MIER1-3A; the name MIER1 refers to variants lacking exon 3A. The cDNAs used in this study are the coding sequences of *mier1α* (GenBank accession no. AY124187), *mier1β* (AY124190), *mier1*-*3Aα* (AY124186) and *mier1-3Aβ* (AY124189).

Alternative splicing to include the cassette exon 3A positions the additional sequence immediately after the second codon in MIER1, located at the end of exon 2A (Fig. 1A). However, this additional sequence shifts the reading frame such that the start of translation for MIER1-3A is predicted to be in exon 3A (Fig. 1A, ‘M’ in blue box) and the alternate N-terminal sequence becomes: MFMFNWFTDCLWTLFLSNYQ- (Fig. 1A).

To confirm the start of translation for this N-terminal isoform, we produced two mutant constructs: in the first, the original MIER1 initiation codon located at the end of exon 2A, as well as a downstream in-frame ATG (Fig. 1A, yellow highlight), were mutated, changing the encoded sequence from MLKM to QLKL. In the second construct, the predicted initiation codon in exon 3A and a downstream ATG that encodes the 3^rd^ residue were altered to produce an MFMF->IFIF mutant. Coupled *in vitro* transcription-translation of the wild-type and mutant cDNAs was performed followed by immunoprecipitation with an anti-MIER1 antibody and the resulting proteins analyzed by SDS-PAGE/fluorography. As can be seen in Fig. 1B, mutating the ATGs in the first construct did not prevent translation of full–length MIER1-3A protein (compare lanes 1 and 3 in Fig. 1B), while mutating the predicted initiation codon for MIER1-3A completely abolished production of full-length protein (Fig. 1B, lane 2). These data confirm that translation of the *mier1*-*3A* transcript is initiated within exon 3A and encodes the N-terminal sequence: MFMFNWFTDCLWTLFLSNYQ-.

### Exon 3A encodes a classic leucine-rich nuclear export signal

Analysis of the exon 3A amino acid sequence for possible motifs revealed the presence of a putative leucine-rich NES (underlined in Fig. 1A) that conforms to the consensus: φ–x_(2–3)_–φ–x_(2–3)_–φ–x–φ, where φ represents L, I, V, F or M and x is any amino acid [18], [19]. Our previous work demonstrated that the localization of MIER1α changes from nuclear to cytoplasmic during progression to invasive breast carcinoma [7]. Thus, the possibility that alternative splicing could place a potential NES in the MIER1α sequence has important implications for MIER1α's role in breast cancer. Therefore, we investigated the effect of including exon 3A sequence on the subcellular localization of MIER1α, using MCF7 breast carcinoma cells. This cell line retains many of the characteristics of differentiated epithelial cells (reviewed in [20]) and MIER1α is targeted to the nucleus in these cells (unpublished observations).

**Figure 2:**
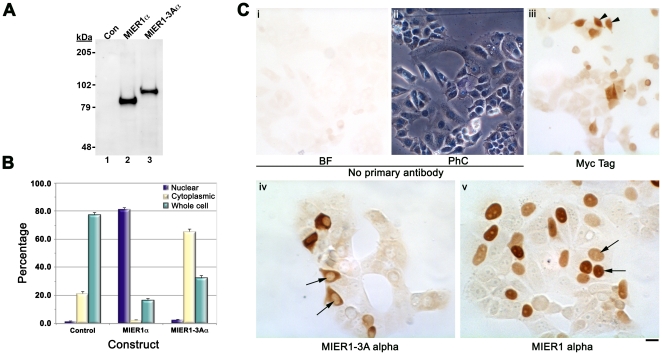
Subcellular localization of the MIER1α and MIER1-3Aα isoforms in MCF7 cells. MCF7 cells were transfected with myc-tagged MIER1α, MIER1-3Aα or empty vector and analyzed by immunocytochemistry or immunoblotting with the 9E10 monoclonal antibody. (A) Western blot of extracts from MCF7 cells transfected with empty vector (lane 1), myc-tagged *mier1α* (lane 2) or myc-tagged *mier1-3Aα* (lane 3); staining was performed with the 9E10 antibody and confirms that a single protein in each cell extract is recognized by the antibody. The positions of the molecular weight standards are indicated on the left. (B) Histogram showing the results of 3 experiments; random fields were selected and the staining pattern of each cell within the field was scored visually according to the categories described in the Results & Discussion. 650–1,350 cells were scored for each construct. Plotted is the percentage of cells in each category ± S.D. (C) Illustrative examples of the observed staining pattern. Panels (i) & (ii) show brightfield (BF) and the corresponding phase contrast (PhC) views of a staining control, prepared without primary antibody. Panel (iii) shows cells expressing the myc-tag alone; examples of whole cell staining are indicated by arrowheads. Panels (iv) & (v) show cells expressing MIER1-3Aα & MIER1α, respectively; note the absence of nuclear staining in panel (iv) while nuclei in panel (v) are intensely stained (arrows). Scale bar = 50 µm for (i)–(iii) and 25 µm for (iv)–(v).

MCF7 cells were transfected with plasmids encoding myc-tagged MIER1α, myc-tagged MIER1-3Aα or myc-tag alone and the localization of MIER1 was determined by immunocytochemistry. The anti-tag antibody (9E10) used for this study recognizes a single protein band on a Western blot of transfected MCF7 cells expressing MIER1α or MIER1-3Aα (Fig. 2A). Cells were scored according to the following pattern of staining: nuclear: the nucleus was intensely stained, with little or no cytoplasmic staining; cytoplasmic: staining was observed throughout the cytoplasm, with little or no staining in the nucleus; whole cell: both the nucleus and cytoplasm were stained. Cells expressing the myc-tag alone displayed primarily whole cell staining, in which the nucleus and cytoplasm were stained with equal intensity (Fig. 2B & 2C, panel iii); this localization pattern was expected since the molecular size of the expressed tag (8.5 kDa) is sufficiently small to allow for passive diffusion between nucleus and cytoplasm [21]. In cells expressing MIER1α, localization was nuclear in 81% of cells (Fig. 2B and 2C, panel v); the remainder of the cells displayed whole cell staining and for most of these, the nucleus was more intensely stained than the cytoplasm. MIER1-3Aα expressing cells, on the other hand, showed virtually no staining that was exclusively nuclear (Fig. 2B). Instead, staining was cytoplasmic in 66% of cells and whole cell in 32% (Fig. 2B and 2C, panel iv), with most of the latter category displaying equal intensity staining in the nucleus and cytoplasm. These data demonstrate that addition of the exon 3A sequence in MIER1α changes its subcellular localization in MCF7 cells.

**Figure 3:**
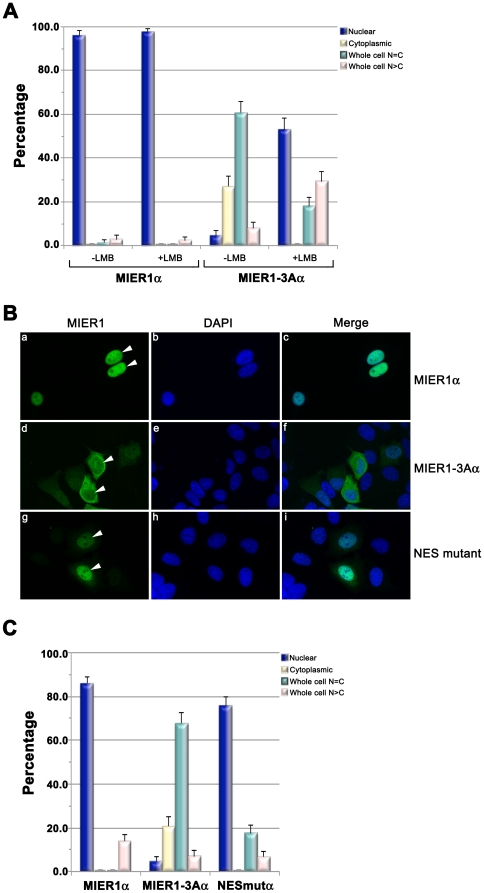
Function of exon 3A sequence in nuclear export. (A) Effect of leptomycin B on localization of MIER1α and MIER1-3Aα. Cells were transfected, treated with 5 ng/ml LMB for 24 h and analyzed by confocal microscopy, using DAPI, 9E10 and a DyLight-488 secondary antibody . Histogram showing the results of 2 experiments; the staining pattern from random fields was scored visually according to the categories described in the Results & Discussion. Plotted is the percentage of cells in each category ± S.D; 75–90 cells were scored for each construct with each treatment. Note the increase in nuclear localization in treated cells expressing MIER1-3Aα. (B) and (C) Mutation of the NES consensus increases nuclear localization. MCF7 cells were transfected with plasmids encoding myc-tagged MIER1α, MIER1-3Aα or MIER1-3Aα containing a double mutation in the NES consensus (NES mutant) and analyzed by confocal microscopy using the antibodies described in (A). (B) Illustrative examples of cells expressing MIER1α (a–c), MIER1-3Aα (d–f) or the NES mutant (g–i); arrowheads indicate nuclei. (C) Histogram showing the results of 2 experiments; the staining pattern was scored as in (A). Plotted is the percentage of cells in each category ± S.D; 85–130 cells were scored for each construct.

To determine whether the localization of MIER1-3Aα in the cytoplasm is due to increased nuclear export rather than inhibition of nuclear import, we examined the effect of leptomycin B (LMB) on the localization pattern of MIER1α and MIER1-3Aα proteins. LMB blocks nuclear export by covalently modifying CRM1 [22], [23], a key receptor in NES-mediated nuclear export (reviewed in [24]), while having no effect on import. Therefore, if the exon-3A sequence functions in nuclear export, one would expect to see an accumulation of MIER1-3Aα in the nucleus of treated cells. MCF7 cells expressing MIER1α or MIER1-3Aα were treated with 5 ng/ml LMB for 24 h and the localization pattern was determined by confocal microscopy. In this and subsequent experiments, the ‘Whole Cell’ staining category was subdivided into two: 1) whole cell N = C, in which the nucleus and cytoplasm were stained with equal intensity, and 2) whole cell N>C, in which the nucleus was much more intensely stained than the cytoplasm. LMB had no effect on the subcellular localization pattern of MIER1α (Fig. 3A). However, for MIER1-3A, the percentage of cells with exclusively nuclear MIER1-3Aα increased from 4% to 53% in the presence of LMB (Fig. 3A). There was also a shift in the pattern of whole cell staining: in the absence of LMB, the majority of cells displayed equal intensity nuclear and cytoplasmic staining, while the majority of LMB-treated cells in this category showed more intense staining in the nucleus than in the cytoplasm. These data demonstrate that the preferential cytoplasmic localization of MIER1-3Aα is due to CRM1-dependent nuclear export.

To confirm that nuclear export of MIER1-3Aα is due to the identified NES, we mutated the consensus sequence. Previous studies have demonstrated that mutating the last 2 hydrophobic residues in the consensus is sufficient to interfere with NES activity [18], [25]. Therefore, we produced a ^14,16^L->A double mutant. MCF7 cells expressing MIER1α, MIER1-3Aα or the NES mutant were scored for subcellular localization by confocal microscopy. Mutating the NES consensus resulted in a significant increase in the percentage of cells with exclusively nuclear staining, from 4% to 76%, with a concomitant decrease in the percentage of cells showing whole cell N = C staining (Fig. 3B–C). Moreover, no exclusively cytoplasmic staining was observed (Fig. 3C). These results provide evidence that the identified NES functions in nuclear export and is responsible for the cytoplasmic localization of the MIER1-3Aα isoform.

### Nuclear localization of MIER1β is not affected by the inclusion of exon 3A sequence

**Figure 4:**
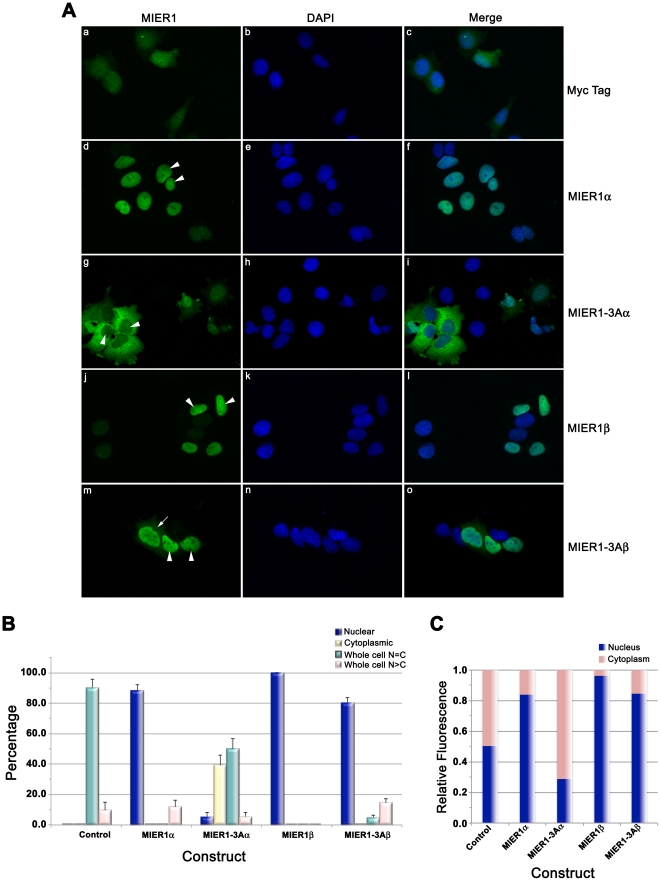
Subcellular localization of the MIER1-3Aβ isoform in MCF7. MCF7 cells were transfected with myc-tagged MIER1α, MIER1-3Aα, MIER1β, MIER1-3Aβ or empty vector and analyzed by confocal microscopy, performed as in the legend to Fig. 3. (A) Illustrative examples of cells expressing myc-tag alone (a–c), MIER1α (d–f), MIER1-3Aα (g–i), MIER1β (j–l) or MIER1-3Aβ (m–o). Arrowheads indicate nuclei and the arrow indicates staining in the cytoplasm. (B) Histogram showing the results of 4 independent experiments; the staining pattern was scored as in the legend to Fig. 3. Plotted is the percentage of cells in each category ± S.D; 55–160 cells were scored for each construct. Note that, unlike MIER1-3Aα, MIER1-3Aβ remains predominantly nuclear. (C) Bar graph showing the intracellular distribution of each construct. Pixel values for the nuclear and in the cytoplasmic areas were measured using Image J v1.46 and plotted as a proportion of the total signal. Shown is the proportion in each compartment, using measurements from 30–40 cells for each construct.

MIER1β contains a strong nuclear localization signal (NLS) in its β-specific C-terminus [5] and in most cells, it is targeted exclusively to the nucleus [1], [5]. Therefore, we investigated whether the inclusion of exon 3A sequence would alter this pattern. MCF7 cells expressing myc-tagged MIER1β, MIER1-3Aβ, MIER1α, MIER1-3Aα or myc-tag alone were analyzed by confocal microscopy (Fig. 4). As expected, the expressed myc-tag alone was distributed throughout the cell (Fig. 4A, panel a-c and 4B) and MIER1β was exclusively nuclear in all cells (Fig. 4A, panel j-l and 4B). Likewise, MIER1α was predominantly nuclear (Fig. 4A, panel d-f and 4B) and very few cells (<6%) expressing MIER1-3Aα displayed nuclear staining (Fig. 4A, panel g–i and 4B). Interestingly, the MIER1-3Aβ localization pattern did not mirror that of MIER1-3Aα; instead, 80% of cells displayed nuclear staining (Fig. 4A, panel m-o and Fig. 4B) and most of the remaining 20% showed whole cell staining, with the nucleus more intensely stained than the cytoplasm (Fig. 4B). Furthermore, none of the cells expressing MIER1-3Aβ showed exclusively cytoplasmic staining (Fig. 4B), as seen with MIER1-3Aα.

To further characterize the localization of the MIER1 isoforms, we performed an analysis of the confocal z-stacks, using an Image J software program to provide a quantitative measure of the fluorescence in the nuclear and cytoplasmic compartments of cells expressing each isoform. The results of this analysis (Fig. 4C) show that for the 3 isoforms: MIER1α, MIER1β and MIER1-3Aβ, more than 80% of the protein is localized in the nucleus, while for MIER1-3Aα, over 70% of the protein is cytoplasmic. Taken together, our results demonstrate that exon 3A functions specifically to shuttle the α isoform from the nucleus to the cytoplasm.

The exon 3A sequence appears to function specifically to regulate nuclear levels of MIER1α. There are numerous examples of transcriptional regulators, whose function is altered in neoplastic cells by changes in nucleo-cytoplasmic distribution. Classic examples include Rb [26], p53 [27] and BRCA1 (reviewed in [28]); the mechanisms responsible are diverse and involve mutation, phosphorylation, monoubiquitylation and alternative splicing (reviewed in [28], [29]). The latter has been described as a regulatory mechanism for the Kruppel-like zinc finger transcription factor 6 (KLF6) [30], [31]. KLF6 functions as a tumour suppressor [32] but a splice variant, KLF6-SV1, truncated at its C-terminus, localizes in the cytoplasm where it acts as a dominant negative to antagonize KLF6 growth suppressive activity [30]. Increased expression of this alternatively spliced oncogenic isoform has been described in a number of cancers, including prostate, colorectal, pancreatic and ovarian (reviewed in [33]).

Our results suggest that controlling differential splicing to alter nuclear levels of MIER1α could represent an important mechanism for regulating its chromatin modifying activities and ultimately gene expression; it might also play a role in the development of invasive breast carcinoma. Given these results, it will be important to examine the expression pattern of MIER1-3Aα in breast tumour samples, once suitable antibodies become available.

## Materials and Methods

### Cell culture

The MCF7 cell line was purchased from the American Tissue Culture Collection and cultured in DMEM (GIBCO) containing 10% fetal calf serum (GIBCO) in a 37°C incubator with 5% CO_2_. To inhibit nuclear export, 5 ng/ml leptomycin B (Sigma-Aldrich Co.) was added to the culture medium for 24 h before fixation for microscopy.

### Plasmids and site-directed mutagenesis

The structure of the human *mier1* gene, the sequence of its transcripts and preparation of myc-tagged MIER1 constructs have been described previously [3]. Primers for mutating the ATGs and the putative NES were designed using Stratagene's QuikChange Primer Design program. Mutagenesis was carried out using Stratagene's QuikChange Lightning kit according to the manufacturer's instructions and the following sets of primers: for MLKM->QLKL, forward and reverse primers were: 5′-CAATGCAGACAAGACGGATGTGCAGCTGAAATTGTGCATCAGATGTTTATGTTT-3′ and 5′-AAACATAAACATCTGATGCACAATTTCAGCTGCACATCCGTCTTGTCTGCATTG-3′ respectively. For MFMF->IFIF, forward and reverse primers were: 5′-ATGCTGAAAATGTGCATCAGATCTTTATCTTTAATTGGTTTACAGACTGTC-3′ and 5′-GACAGTCTGTAAACCAATTAAAGATAAAGATCTGATGCACATTTTCAGCAT-3′, respectively. For the NES mutant, ^14,16^L→A, forward and reverse primers were: 5′-GGTTTACAGACTGTCTGTGGACTGCTTTCGCGTCAAATTACCAGCCATCTGTTG-3′ and 5′-CAACAGATGGCTGGTAATTTGACGCGAAAGCAGTCCACAGACAGTCTGTAAACC-3′, respectively. Myc-tagged MIER1-3A constructs were produced by PCR cloning full-length sequences into the BglII site of the CS3+MT plasmid as described in [3], using the forward primer: 5′-CGGGATCCAGATGTTTATGTTTAATTGGTTTACA-3′ and either 5′- CGGGATCCAAAACAAGACCACAGAAGC-3′ (alpha) or 5′-CTTGAAAACACAGATGACTAAGGATCCCG-3′ (beta) reverse primers. All plasmids were prepared using Clontech's NucleoBond Endotoxin-free Maxi Plasmid kit and the sequences/mutations were confirmed by automated dideoxynucleotide sequencing of both strands (performed by The Centre for Applied Genomics, The Hospital for Sick Children, Toronto, Canada).

### *In vitro* coupled transcription/translation, immunoprecipitation and Western Blotting

^35^S-labelled MIER1 was synthesized using a coupled *in vitro* transcription/translation system (Promega Corp.) and subjected to immunoprecipitation, as previously described [34], with the following modifications: 2 µl of translation products were immunoprecipitated using 10 µl of an anti-MIER1 antibody [3] that recognizes a sequence in the common internal region; immunoprecipitated proteins were analyzed by SDS-PAGE followed by fluorography as in [34].

Western blot analysis was performed as in [34], using 60 µg cellular protein per lane. Transfers were performed onto Hybond-P PVDF membranes (GE Healthcare Corp.); the membranes were stained using a 1∶1000 dilution of 9E10 monoclonal antibody, 1∶3000 HRP-labelled sheep anti-mouse antibody and Amersham's ECL Western Blotting System (GE Healthcare Corp.).

### Transient Transfection

Cells were transfected according to the manufacturers' protocol using either the Mirus TransIT-LT1 transfection reagent (Medicorp, Inc.) and a 3∶1 ratio of reagent:DNA (v/w), or by electroporation using the Neon system (Invitrogen Corp.) and the following parameters: 1250 V, 20 ms, 2 pulses. Eighteen hours prior to transfection with the TransIT-LT1 reagent, cells were plated at a density of 2×10^4^/well into Falcon 8-well culture slides (BD BioSciences) and all transfections were performed using 0.26 µg of plasmid. For electroporation, 3×10^5^ cells and 0.5 µg of plasmid were loaded into a 10 µl tip; after transfection, cells were dispensed at a density of 2×10^4^/well into 8-well culture slides for immunocytochemistry (ICC)/confocal or at a density of 3×10^5^ in 35 mm dishes for Western blot (WB) analysis. Transfected cells were cultured for 48 h, then either fixed in 4% paraformaldehyde/PBS (ICC) or solubilized in 500 µl SDS-PAGE sample buffer (WB).

### Immunocytochemistry, Confocal Microscopy and Image Analysis

After fixation, cells were processed for either immunocytochemistry (ICC) as described previously [35] or for confocal microscopy as described in [36], using the 9E10 anti-myc monoclonal antibody, prepared as in [10] and used at a 1∶200 dilution. Cells were incubated with primary antibody overnight at 4°C. For ICC, cells were incubated with a 1∶200 dilution of HRP-labelled sheep anti-mouse antibody (GE Healthcare Corp.) for 1 h and stained using SigmaFast 3,3′-Diaminobenzidine (DAB) (Sigma-Aldrich Co.), prepared according to the manufacturer's instructions. For confocal analysis, a DyLight-488 labeled donkey anti-mouse secondary antibody (Jackson ImmunoResearch Laboratories, Inc.) was utilized; the antibody was re-constituted according to the manufacturer's instructions and used at a 1∶250 dilution. Nuclei were counterstained using 2.5 µg/ml 4′,6-diamidino-2-phenylindole (DAPI; Sigma-Aldrich Co.). All slides were mounted in 10% glycerol/PBS. Brightfield and phase contrast images were captured using an Olympus BH-2 microscope equipped with a CoolSnap digital camera. Fluorescence images were acquired using sequential Z-stage scanning in two channels (DAPI and DyLight488) on an Olympus FluoView FV1000 confocal microscope; Z-stacks were compiled into individual images.

Quantitative analysis of confocal z-stacks was performed using Image J software v1.46 [37] as follows: using random fields, cell outlines from the projected z-stacks were traced, the sum of the pixel values within the outlines in the MIER1 channel was determined and the background was subtracted; this value was used to represent MIER1 fluorescence within the whole cell. The nuclei were outlined and the sum of the pixel values was obtained and the background subtracted; this value was used as MIER1 fluorescence within the nucleus. The nuclear value was subtracted from the whole cell value to obtain that representing MIER1 fluorescence in the cytoplasm. For each construct, 30–40 cells were measured from 4 independent experiments.
